# Perseverance and consistency of interest in underrepresented post-doctoral fellows and early-career faculty

**DOI:** 10.1017/cts.2023.523

**Published:** 2023-04-13

**Authors:** Maya S. Thakar, Chantele Mitchell-Miland, Natalia E. Morone, Andrew D. Althouse, Audrey J. Murrell, Doris M. Rubio, Gretchen E. White

**Affiliations:** 1 Institute for Clinical Research Education, University of Pittsburgh Schools of the Health Sciences, Pittsburgh, Pennsylvania, USA; 2 General Internal Medicine, Boston University School of Medicine, Boston, Massachusetts, USA; 3 Boston Medical Center, Boston, Massachusetts, USA; 4 University of Pittsburgh, School of Medicine, Pittsburgh, Pennsylvania, USA; 5 College of Business Administration, University of Pittsburgh, Pittsburgh, Pennsylvania, USA

**Keywords:** Grit, diversity equity inclusion, career development, diversifying the biomedical research workforce, science identity, burnout

## Abstract

**Introduction:**

Underrepresented researchers face more challenges than their well-represented counterparts. Perseverance and consistency of interest are associated with career success in well-represented physicians. Therefore, we examined associations of perseverance and consistency of interest with Clinical Research Appraisal Inventory (CRAI), science identity, and other factors related to career success among underrepresented post-doctoral fellows and early-career faculty.

**Methods:**

This is a cross-sectional analysis of data collected from September to October 2020 among 224 underrepresented early-career researchers at 25 academic medical centers in the Building Up Trial. We used linear regression to test associations of perseverance and consistency of interest scores with CRAI, science identity, and effort/reward imbalance (ERI) scores.

**Results:**

The cohort is 80% female, 33% non-Hispanic Black, and 34% Hispanic. The median perseverance and consistency of interest scores were 3.8 (25th–75th percentile: 3.7,4.2) and 3.7 (25th–75th percentile: 3.2, 4.0), respectively. Higher perseverance was associated with a higher CRAI score (*β* = 0.82; 95% CI = 0.30, 1.33, *p* = 0.002) and science identity (*β* = 0.44; 95% CI = 0.19, 0.68, *p* = 0.001). Higher consistency of interest was associated with a higher CRAI score (*β* = 0.60; 95% CI = 0.23, 0.96, *p* = 0.001) and higher science identity score (*β* = 0.20; 95% CI = 0.03, 0.36, *p* = 0.02), while lower consistency of interest was associated with imbalance favoring effort (*β* = –0.22; 95% CI = –0.33, –0.11, *p* = 0.001).

**Conclusions:**

We found that perseverance and consistency of interest are related to CRAI and science identity, indicating that these factors may positively influence one’s decision to stay in research.

## Introduction

Perseverance and consistency of interest for long-term goals are collectively known as grit, and predict goal achievement, pursuit of higher education, and career success [1]. Career advancement and career success are influenced by professional self-efficacy and science identity in researchers from the UK [2]. People with stronger professional self-efficacy, or the confidence in one’s ability to succeed in their career, are more likely to persist in that career despite obstacles that arise. Similarly, science identity, or a person’s sense of self in relation to their scientific knowledge and competence as well as whether they are welcomed and seen as a scientist [3], is directly related to pursuit of and persistence in a science career [4,5,6]. However, research examining the association between grit and professional self-efficacy is limited. One study found that grit and professional self-efficacy were positively correlated in university professors in the Phillippines [7]. To our knowledge, the relationship between grit and science identity has not been evaluated.

Higher levels of perseverance and consistency of interest were associated with lower levels of psychological distress in Chinese adults [8]; however, the relationship between perseverance, consistency of interest, and other indicators of professional distress (i.e., effort-reward imbalance [ERI] and burnout) is not well understood. Scientists experience ERI when they exert more effort in their careers than the amount of reward they receive in terms of salary, career promotion, job security, and esteem or recognition; leading to frustration and distress [9]. It is unclear whether higher perseverance and consistency of interest mitigate ERI. Burnout consists of overwhelming exhaustion, separating oneself from working with colleagues, and low levels of professional self-efficacy [10]. In well-represented residents and faculty physicians, higher levels of grit were associated with lower levels of burnout [11,12].

The lack of racial and ethnic diversity in the biomedical research workforce and the disproportionate rate at which underrepresented (UR) biomedical researchers and faculty leave research positions are well-documented issues [13–16]. UR researchers face more challenges than their well-represented counterparts; for this reason, it is important to develop future interventions to retain UR scientists in biomedical research [16]. Many factors, such as personal experiences with racism, historically rooted stereotypes, and lack of representation in biomedical research, negatively impact one’s professional self-efficacy and identity in UR scientists [17–19].

While perseverance, consistency of interest, professional self-efficacy, and science identity are all related to career success, prior literature fails to examine these relationships in UR populations. Therefore, we examined associations of perseverance and consistency of interest with Clinical Research Appraisal Inventory (CRAI), science identity, effort–reward imbalance, and burnout, which may be related to career success, among 224 post-doctoral fellows and early-career faculty who are underrepresented in health-related sciences from 25 institutions. Because many physicians experienced psychological distress during the COVID-19 pandemic that was uniquely related to patient care [20], we further examined the relationships stratified by the highest degree obtained (physician-scientists versus PhD researchers).

## Methods

### Design and Participants

Building Up is a cluster-randomized trial of 224 post-doctoral fellows and early-career faculty (i.e., within five years of first academic appointment) who are underrepresented in health-related sciences (i.e., people from racial or ethnic groups underrepresented in health-related science, people with disabilities, people from disadvantaged background, or are women) according to the National Institutes of Health definition. [21,22]. The trial is conducted at 25 academic institutions and includes two intervention arms with varying intensity of four intervention components (i.e., monthly sessions, networking, coursework, and mentoring) aimed at addressing the issue of the leaky career pathway for UR faculty in biomedical research. The institutions were diverse; including public (University of Wisconsin, Madison) and private (Rush University Medical Center) institutions, large (University of Michigan) and small (University of Chicago) institutions, and those with limited (Penn State Health) and more diversity (University of Southern California) (Supplemental Table 1). A single Institutional Review Board at the University of Pittsburgh approved the protocol. Participants provided electronic informed consent, were informed that their responses were confidential, and were informed that there would be 4 (i.e., 1 pre-intervention plus 3 annual post-intervention) survey-based assessments.

Participant recruitment has been previously described [22]. Participants electronically completed pre-intervention assessments in September-October 2020. Pre-intervention data for participants in both intervention arms were included in this analysis.

### Measures

Participants were asked to report gender, race and ethnicity, and degrees achieved. Due to small numbers, a non-Hispanic “other” race/ethnicity category was created that included the American Indian or Alaskan Native, Middle Eastern or North African, Native Hawaiian or Other Pacific Islander, or other categories. Physician-scientists are participants with an MD as their highest degree and PhD researchers are those with a PhD as their highest degree. “Other” highest degree achieved included MD/PhD, PharmD, PsyD, DDS/DMD, DVM, or other.

The 12-item Grit Scale is a validated questionnaire designed to measure grit [23]. The scale consists of two 6-item components: perseverance (items 1, 4, 5, 6, 10, and 12) and consistency of interest (items 2, 3, 5, 7, 8, and 11). Each item is measured using a 5-point Likert scale ranging from 1 (“not like me at all”) to 5 (“very much like me”). The minimum score on each scale is 1 (not at all gritty) and the maximum score is 5 (extremely gritty). Scores were not calculated if any items on the scales were missing.

The Clinical Research Appraisal Inventory-12 (CRAI-12) is a validated and reliable 12-item scale designed to assess the self-confidence of trainees in performing clinical research [24]. Respondents were asked to rank their confidence in performing each clinical research task on a scale of 0 (indicating no confidence to perform the task successfully today) to 10 (indicating total confidence). Responses were summed and averaged for a total CRAI-12 score ranging from 0 (no self-confidence) to 10 (high levels of self-confidence).

Science Identity is a 5-item questionnaire measuring how much participants think being a scientist is part of their personal identity. Participants rated each item using a 5-point Likert scale ranging from 1 (“strongly disagree”) to 5 (“strongly agree”). Responses were summed and averaged for a total science identity score ranging from 1 (no science identity) to 5 (strong science identity).

The Effort Reward Imbalance (ERI) questionnaire measures effort, reward, and over-commitment to determine the presence of ERI [25]. Participants rated each of the 10 items using a 5-point Likert scale ranging from 1 (“does not apply”) to 5 (“does apply and very strained”). Three items measure effort, and 7 items measure reward. Scoring has been previously described [26]. ERI < 1 indicates an imbalance that favors rewards in which participants exert less effort and receive more rewards. ERI > 1 indicates an imbalance that favors effort; participants exert more effort and receive less rewards [26].

Using a validated single-item measure of burnout [27], participants were asked to select their level of burnout as “I enjoy my work. I have no symptoms of burnout,” “Occasionally, I am under stress. I don't always have as much energy as I once did, but I don't feel burned out,” “I am definitely burning out and have one or more symptoms of burnout, such as physical and emotional exhaustion,” “The symptoms of burnout that I am experiencing won't go away. I think about frustration at work a lot,” and “I feel completely burned out and often wonder if I can go on. I am at the point where I may need some changes or may need to seek some sort of help. [27]” Because responses to the last two options were rare, these response options were collapsed into one category. We further dichotomized burnout as having no symptoms of burnout (i.e., “I enjoy my work. I have no symptoms of burnout” or “Occasionally, I am under stress. I don't always have as much energy as I once did, but I don't feel burned out”) and having symptoms of burnout (i.e., “I am definitely burning out and have one or more symptoms of burnout, such as physical and emotional exhaustion,” “The symptoms of burnout that I am experiencing won't go away. I think about frustration at work a lot,” and “I feel completely burned out and often wonder if I can go on. I am at the point where I may need some changes or may need to seek some sort of help”) [27].

### Statistical Analysis

SAS version 9.4 (SAS Institute, Cary, NC, USA) was used for all analyses. Reported *p*-values are two-tailed; a *p*-value < 0.05 was significant. As this was predominantly an exploratory cross-sectional analysis, we did not account for multiple comparisons [28].

Participant characteristics are reported as medians and 25^th^ and 75^th^ percentiles for continuous data and frequencies and percentages for categorical data. Characteristics of physician-scientists and PhD researchers were compared with Kruskal–Wallis tests for continuous data and chi-square tests for categorical data.

A series of regression models were used to test and estimate independent associations of perseverance and consistency of interest with clinical research appraisal, science identity, ERI, and burnout. Linear regression was used for clinical research appraisal, science identity, and ERI. Logistic regression was used for burnout. First, the association between perseverance and clinical research appraisal, science identity, ERI, and burnout were evaluated in separate models. Then this was repeated for consistency of interest. Next, the independent associations of perseverance and consistency of interest, with adjustment for age, race/ethnicity, and gender, were assessed for each outcome (i.e., clinical research appraisal, science identity, ERI, and burnout) [1,29]. Because many physicians experienced psychological distress during the COVID-19 pandemic that was uniquely related to patient care [20], we stratified all analyses by highest degree obtained (physician-scientists versus PhD researchers).

## Results

**Fig. 1.**
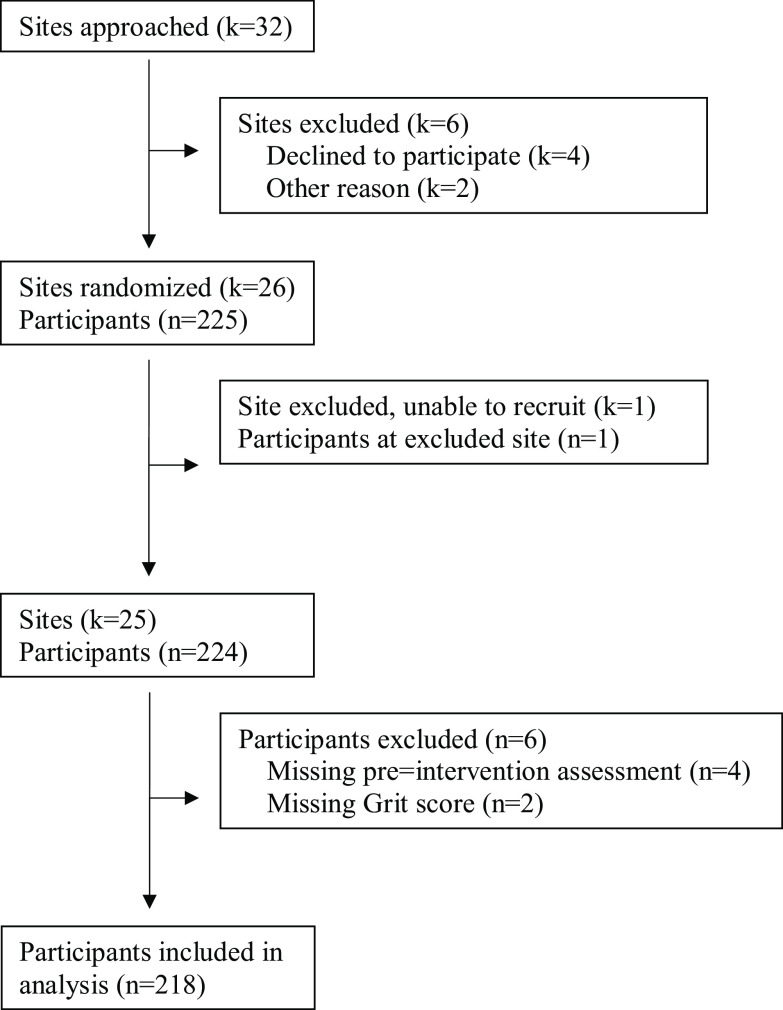
Institution and participant flow diagram for the building up a diverse biomedical research workforce trial.

Ninety-seven percent (218/224) of participants completed the pre-intervention Grit Scale and were included in analysis (Fig. 1). Characteristics of participants are described in Table 1. The cohort is 80% female, 34% Hispanic/Latinx, and 33% non-Hispanic/Latinx Black. Fifty-nine percent (*n* = 127) were PhD researchers, 32% (*n* = 69) were physician-scientist, and 8% (*n* = 21) had “other” highest degrees. The median age was 37 years (25^th^–75^th^ percentile: 34–41). The median overall grit score was 3.8 (25^th^–75^th^ percentile: 3.5–4.3), which translates to “very gritty.” The median perseverance score was 3.8 (25^th^–75^th^ percentile: 3.7–4.2) and the median consistency-of-interest score was 3.7 (25^th^–75^th^ percentile: 3.2–4.0). Physician-scientists and PhD researchers were similar with the following exceptions. Physician-scientists, when compared to PhD researchers, were significantly more years since graduating with their highest degree (9 years versus 4 years, *p* < .001), had a lower median CRAI-12 score (6.0 versus 6.6, *p* = 0.01) and a lower median science identity score (3.6 versus 4.2, *p* < .001).

**Table 1.** Characteristics of underrepresented post-doctoral fellows and early-career faculty, Building Up a Diverse Biomedical Research Workforce trial

	No. (%)^a^			
Characteristic	Total (No.=218)	Physician Scientist (No.=69)	PhD Researcher (No.=127)	*P*
Age (median, 25^th^–75^th^ percentile)	37 (34–41)	36 (33–38)	36 (32–40)	0.76
Gender				0.27
Male	43 (19.8)	9 (13.2)	27 (21.3)	
Female	173 (79.7)	59 (86.8)	126 (78.0)	
Gender minority	1 (0.5)	0 (0.0)	1 (0.79)	
Race/ethnicity				0.94
Hispanic/latinx	75 (34.4)	25 (36.8)	42 (32.8)	
Non-hispanic/latinx				
White	29 (13.3)	8 (11.8)	19 (14.8)	
Black	72 (33.0)	21 (30.9)	41 (32.0)	
Asian	26 (11.9)	8 (11.8)	17 (13.3)	
Other and multi-racial	16 (7.3)	6 (8.8)	9 (7.0)	
Years since highest degree achieved (median, 25^th^–75^th^ percentile)	5(3–9)	9 (5–12)	4 (2–7)	< 0.001
Range	0–28	2–24	0–17	
Grit (median, 25^th^–75^th^ percentile)	3.8 (3.5–4.3)	3.9 (3.5–4.2)	3.8 (3.4–4.3)	0.98
Range	2.3–5.0	2.3–4.7	2.6–5.0	
Perseverance (median, 25^th^75^th^ percentile)	3.8 (3.7–4.2)	3.8 (3.6–4.0)	4.0 (3.5–4.2)	0.09
Range	2.5–4.8	2.8–4.5	2.5–4.8	
Consistency of interest (median, 25^th^–75^th^ percentile)	3.7 (3.2–4.0)	3.7 (3.2–4.0)	3.7 (3.1–4.1)	0.68
Range	1.3–5.0	1.3–4.7	1.3–5.0	
Clinical research appraisal inventory-12 (median, 25^th^–75^th^ percentile)	6.4 (5.5–7.3)	6.0 (5.3–6.5)	6.6 (5.6–7.7)	0.01
Range	2.8–9.8	2.8–9.8	3.3–9.8	
Science identity (median, 25^th^–75^th^ percentile)	4.0 (3.4–4.6)	3.6 (3.2–4.4)	4.2 (3.6–4.6)	< 0.001
Range	1.0–5.0	1.8–5.0	1.0–5.0	
Effort reward imbalance (median, 25^th^–75^th^ percentile)	1.0 (0.7–1.3)	1.0 (0.7–1.3)	0.8 (1.0–1.3)	0.25
Range	0.3–4.4	0.3–1.9	0.3–4.4	
Burnout				0.92
No symptoms	21 (9.6)	6 (8.8)	14 (10.9)	
Occasionally under stress. Not as much energy as once has but don't feel burned out	107 (49.1)	33 (48.5)	63 (49.2)	
Definitely burning out and have more than one symptom	77 (35.3)	25 (36.8)	42 (32.8)	
Symptoms of burnout won't go away/completely burned out and wonder if can go on	13 (6.0)	4 (5.9)	9 (7.0)	

a Unless otherwise specified. The number of participants across categories may not sum to the total due to missing data.

Results from unadjusted and adjusted models for the overall sample are summarized in Table 2. After adjustment for consistency of interest and other covariates, each one-point increase in perseverance score was associated with a 0.82 point higher CRAI-12 score (*β* = 0.82; 95% CI = 0.30, 1.33, *p* = 0.002) and 0.44 point higher science identity score (*β* = 0.44; 95% CI = 0.19, 0.68, *p* = 0.001). After adjustment for perseverance, age, gender, and race/ethnicity, higher consistency of interest was significantly associated with higher CRAI-12 score (*β* = 0.60; 95% CI = 0.23, 0.96, *p* = 0.001) and stronger science identity (*β* = 0.20; 95% CI = 0.03, 0.36, *p* = 0.02), while lower consistency of interest was significantly associated with higher ERI score (*β* = –0.22; 95% C = –0.33, –0.11, *p* = 0.001), reflecting imbalance favoring effort. Higher consistency of interest was significantly associated with lower odds of burnout in unadjusted models (OR = 0.40; 95% CI = 0.17, 0.91, *p* = 0.03) but not in adjusted models (OR = 0.71; 95% CI = 0.47, 1.08, *p* = 0.11).

**Table 2.** Associations of perseverance and consistency of interest with research characteristics, among underrepresented post-doctoral fellows and early-career researchers

	Perseverance				Consistency of interest			
	Unadjusted		Adjusted^a^		Unadjusted		Adjusted^b^	
	*β* (95% CI)	*P*	*β* (95% CI)	*P*	*β* (95% CI)	*P*	*β* (95% CI)	*P*
Clinical research appraisal inventory (CRAI)	0.78 (0.29, 1.27)	0.002	0.82 (0.30, 1.33)	0.002	0.53 (0.21, 0.86)	0.002	0.60 (0.23, 0.96)	0.001
Science identity	0.41 (0.17, 0.65)	0.001	0.44 (0.19, 0.68)	0.001	0.20 (0.04, 0.36)	0.01	0.20 (0.03, 0.36)	0.02
Effort reward imbalance (ERI)	0.03 (–0.13, 0.19)	0.72	0.04 (–0.13, 0.21)	0.65	−0.19 (–0.29, –0.10)	< 0.001	−0.22 (–0.33, –0.11)	<0.001
								
	OR (95% CI)	*P*	OR (95% CI)	*P*	OR (95% CI)	*P*	OR (95% CI)	*P*
Burnout symptoms (ref.=no symptoms)	0.45 (0.15, 1.35)	0.15	0.92 (0.50, 1.73)	0.80	0.40 (0.17, 0.91)	0.03	0.71 (0.47, 1.08)	0.11

OR, odds ratio.

a Adjusted for consistency of interest, age, gender, and race/ethnicity.

b Adjusted for perseverance, age, gender, and race/ethnicity.

Unadjusted and adjusted models stratified by physician-scientists and PhD researchers are summarized in Table 3. Among physician-scientists, after adjustment for covariates including consistency of interest, higher perseverance was significantly associated with higher CRAI-12 score (*β* = 1.57; 95% CI = 0.64, 2.49, *p* = 0.002) and higher science identity score (*β* = 0.60; 95% CI = 0.19, 1.01, *p* = 0.005). After adjustment for perseverance and other covariates, consistency of interest was significantly associated with stronger science identity (*β* = 0.27; 95% CI = 0.004, 0.55, *p* = 0.05) in physician-scientists. Among PhD researchers, after adjustment for covariates, higher perseverance was significantly associated with stronger science identity (*β* = 0.32; 95% CI = 0.02, 0.62, *p* = 0.04) and higher consistency of interest was significantly associated with a higher CRAI-12 score (*β* =0.58; 95% CI = 0.10, 1.05, *p* = 0.02); additionally, higher consistency of interest was significantly associated with a lower ERI score (*β* =–0.29, 95% CI = –0.44, –0.14, *p* < .001).

**Table 3.** Associations of perseverance and consistency of interest with research characteristics among underrepresented post-doctoral fellows and early-career researchers, stratified by physician-scientists and PhD researchers

	Perseverance				Consistency of interest			
	Unadjusted		Adjusted^a^		Unadjusted		Adjusted^b^	
Physician scientist	β (95% CI)	*P*	β (95% CI)	*P*	β (95% CI)	*P*	β (95% CI)	*P*
Clinical research appraisal inventory (CRAI)	1.41 (0.56, 2.25)	0.002	1.57 (0.64, 2.49)	0.002	0.70 (0.15, 1.26)	0.01	0.52 (–0.06, 1.10)	0.08
Science identity	0.59 (0.17, 1.01)	0.01	0.60 (0.19, 1.01)	0.01	0.24 (–0.04, 0.52)	0.09	0.27 (0.004, 0.55)	0.05
Effort reward imbalance (ERI)	−0.08 (–0.32, 0.17)	0.53	−0.09 (–0.35, 0.18)	0.53	−0.09 (–0.25, 0.06)	0.23	−0.08 (–0.25, 0.09)	0.33
	**OR (95% CI)**	***P***	**OR (95% CI)**	***P***	**OR (95% CI)**	***P***	**OR (95% CI)**	***P***
Burnout symptoms (ref.=no symptoms)	0.41 (0.05, 3.43)	0.41	0.70 (0.23, 2.13)	0.53	0.85 (0.23, 3.19)	0.81	1.06 (0.51, 2.20)	0.88
**PhD researcher**	**β (95% CI)**	***P***	**β (95% CI)**	***P***	**β (95% CI)**	***P***	**β (95% CI)**	***P***
Clinical research appraisal inventory (CRAI)	0.45 (–0.15, 1.05)	0.14	0.58 (–0.08, 1.23)	0.09	0.44 (0.04, 0.83)	0.03	0.58 (0.10, 1.05)	0.02
Science Identity	0.26 (–0.02, 0.55)	0.07	0.32 (0.02, 0.62)	0.04	0.19 (0.01, 0.37)	0.04	0.19 (–0.01, 0.39)	0.07
Effort reward imbalance (ERI)	0.06 (–0.15, 0.28)	0.55	0.05 (–0.17, 0.27)	0.65	−0.24 (–0.37, –0.11)	< 0.001	−0.29 (–0.44, –0.14)	< 0.001
	**OR (95% CI)**	***P***	**OR (95% CI)**	***P***	**OR (95% CI)**	***P***	**OR (95% CI)**	***P***
Burnout symptoms (ref.=no symptoms)	0.45 (0.12, 1.70)	0.24	0.97 (0.44, 2.13)	0.94	0.28 (0.10, 0.80)	0.02	0.59 (0.34, 1.00)	0.05

OR, odds ratio.

a Adjusted for consistency of interest, age, gender, and race/ethnicity.

b Adjusted for perseverance, age, gender, and race/ethnicity.

## Discussion

This study is one of the first to examine the relationship between perseverance, consistency of interest, CRAI, science identity, ERI, and burnout among UR post-doctoral fellows and early-career faculty. We found that higher consistency of interest and perseverance were significantly associated with stronger science identity, while lower consistency of interest was significantly associated with putting in more professional effort for less reward. The highest degree achieved by UR early-career biomedical researchers in this sample affected the association between grit subscales and outcome measures.

Our findings indicate that both grit subscales are associated with a stronger sense of science identity among UR post-doctoral fellows and early-career faculty. These findings may have several implications. Having more perseverance and consistency of interest may allow one to be more creative and resourceful about developing their science identity. A longitudinal study is needed to understand how individuals develop perseverance and consistency of interest and how that informs the development of science identity. Likewise, these findings suggest that perseverance and consistency of interest may act as a “buffer” for the harmful impact of negative experiences especially for UR faculty and researchers. While the full impact of these negative experiences is not well understood and is outside the scope of this manuscript, as part of Building Up we conducted qualitative interviews with participants that may further elucidate these negative experiences and their impact. For example, research on the psychological contract involves an individual’s perception of the reciprocal relationship that exists between them and the organization. Within this research, supportive relationships such as mentoring can help individuals to recognize and interpret changes, or what the research calls a “breach” in the psychological contract. These breaches in the psychological contract are known to have a negative impact on key outcomes such as satisfaction, commitment, and retention. Thus, mentoring may be a specific solution to provide the “buffer” for negative experiences for US faculty and researchers [30]. Furthermore, future research should identify whether UR early-career scientists experience more ERI than their well-represented counterparts as this information would be valuable in identifying interventions to retain UR scientists in the biomedical research workforce.

It is difficult to compare our findings to previous studies due to our study taking place at the beginning of the COVID-19 pandemic and during the Racial Justice Movement that was sparked in response to the murder of George Floyd. The median grit score in our full study sample was 3.8, which is similar to the median grit score found in a large sample of American adults from the general population [1]. This is not surprising considering participants in our study are UR and were disproportionately affected by the Racial Justice Movement [31] and COVID-19 [32]. UR post-doctoral fellows and early-career faculty cited no support from institutions, social isolation from colleagues, and discrimination in the workplace following the Racial Justice Movement [31]. Due to the increase in healthcare demands and negative economic impact that COVID-19 caused in institutions across the nation, efforts to foster UR researchers in the biomedical workforce were not a priority [30]. This further widened the disparities that UR researchers face in the biomedical workforce [30]. Grit scores in UR biomedical researchers should be reassessed further out from COVID-19.

When examining grit among physicians alone, the median grit score among physician-scientists (3.9) in our study is higher than the mean grit score in a cohort of 546 physicians in Idaho (mean grit score = 3.3) [33] and a nationally representative sample of 7,464 general surgery residents in the USA (mean grit score = 3.7) [34]. The differences between our findings and those in these studies may be due to different populations. For example, the other studies were not explicitly completed in UR researchers. The higher scores in our cohort may be due to complex sociocultural factors [35] where individuals from UR backgrounds require more grit than their well-represented counterparts to achieve a similar amount of success because they are faced with stereotypes, systemic discrimination, and lack of representation in the biomedical workforce [16,36]. Furthermore, findings from the nationally representative sample of general surgery residents showed that grit scores were significantly higher among female than male surgical residents [34]. This may partially explain the higher grit score in our cohort, which is predominately female.

Among our sample, 40% of participants experienced burnout. This is within the range found in previous studies (33–74%) [37,38]. These findings are surprising because data were collected during the COVID-19 pandemic when levels of burnout were universally high among healthcare professionals [39,40]. Furthermore, because UR researchers were disproportionately affected by the COVID-19 pandemic [30], it’s likely that burnout was higher in UR researchers compared to well-represented researchers. Perseverance and consistency of interest were not significantly associated with burnout in physician-scientists or in PhD researchers in adjusted models. Our results do not confirm those from other studies [41,11,42,38] that more grit is associated with less burnout [38,43]. It is unclear why our results differ from previous studies. To our knowledge, this is one of the first studies to examine the relationship between perseverance, consistency of interest, and burnout in UR early-career scientists. Because the Building Up trial is designed to remeasure participants for up to 3 years following the start of the intervention, we will be able to examine the association between perseverance and consistency of interest and burnout in UR researchers when burnout levels are not inflated by universal stressors.

This study lays a foundation for future researchers to further explore ways in which perseverance and consistency of interest may be related to diversification efforts in the biomedical workforce. The National Institutes of Health has devoted resources to diversifying the workforce in biomedical sciences; however, diversity remains low. A diverse workforce has been shown to boost creativity, problem-solving, productivity, and the quality of learning in students studying at higher education institutions as compared to non-diverse workforces. Increasing diversity in the biomedical workforce can have astounding benefits for productivity and collaboration in the biomedical sciences and has the potential to strengthen the purpose and passion of UR researchers.

Understanding the relationship between perseverance and consistency of interest with professional self-efficacy in UR researchers can help develop evidence-based practices to preserve UR researchers in biomedical research positions. Furthermore, investment in diverse mentoring relationships (e.g., traditional hierarchical, group and peer mentoring) can not only provide support for UR faculty but also act as a buffer for the negative impact of factors such as burnout [44]. Diverse mentoring relationships can also provide the mentoring function of role modeling to support further development of science identity in UR researchers [44]. Effective mentors can help with the recognition, interpretation, and coping behaviors that can produce more resilience and retention among UR faculty [44]. Our findings point to the need for greater investment in organization-sponsored mentoring that can provide both direct and indirect career and psychosocial needs for UR faculty and researchers as they navigate their professional development [44].

Our study had several limitations. Data were collected during the COVID-19 pandemic and the Racial Justice Movement which may have affected our results. Our study was cross-sectional so we cannot conclude that higher perseverance and consistency of interest increase CRAI. It will be important to examine perseverance and consistency of interest and these associations longitudinally to see if the associations change over time. We do not have a comparison group of non-UR post-doctoral fellows and early-career faculty to compare these findings to. Due to our recruitment strategies [22], this sample also has a large proportion of women; reflecting that the National Institutes of Health encourages women to participate in programs designed for recruitment, retention, and career development [21]. This gender disparity in participation should be addressed by future research. There is potential for common source bias due to all the variables being collected in the same survey. There is no statistical method to account for this bias; however, this study is hypothesis-generating and therefore still significantly adds to existing literature.

This study also had several strengths. This study included a large sample of UR post-doctoral fellows and early-career faculty across 25 institutions which increases the generalizability of these findings. We were also able to examine differences among individuals by highest degree achieved which has not been previously done. Most importantly, we were able to contribute to gaps in the literature by examining the association between grit subscales, science identity, and professional psychological distress scales.

## Conclusions

In this study of UR post-doctoral researchers and early-career faculty, we found that higher perseverance and consistency of interest were significantly associated with stronger science identity. We found that these associations differed for physician-scientists and PhD researchers. The results of this study provide a significant contribution that can be built upon to better understand how grit subscales are related to science identity in UR researchers. Future research should aim to understand how perseverance, consistency of interest, CRAI, and science identity are related to career advancement, differ between non-UR and UR researchers, and be used to develop researchers’ purpose and passion for their work. Understanding these factors may lead to developing new interventions to support early-career UR researchers.

## Supplementary material

For supplementary material accompanying this paper visit https://doi.org/10.1017/cts.2023.523. click here to view supplementary material
