# Supplementary material for: Perseverance and consistency of interest in underrepresented post-doctoral fellows and early-career faculty
Source: J Clin Transl Sci. 2023 Apr 13;7(1):e100. doi: 10.1017/cts.2023.523 (PMC10225262; doi:10.1017/cts.2023.523)
Supplement: Supplementary file 1 [file S205986612300523Xsup001.docx]

**Supplemental Table 1**. Institutions involved in Building Up

| Albert Einstein College of Medicine/Montefiore Medical Center |
| --- |
| Children’s National Medical Center/The George Washington University |
| Loyola University Medical Center |
| Massachusetts General Hospital |
| Mayo Clinic Rochester |
| Medical University of South Carolina |
| Northwestern University |
| Oregon Health and Science University |
| Penn State Health |
| Rush University Medical Center |
| Texas A&M University |
| Tufts University Health Sciences |
| University of Buffalo |
| University of California, Davis |
| University of Chicago |
| University of Colorado Denver Anschutz Medical Campus |
| University of Michigan |
| University of Minnesota |
| University of Pennsylvania Perelman School of Medicine |
| University of Southern California |
| University of Texas Health Science Center at San Antonio/University of Texas at Austin |
| University of Virginia |
| University of Wisconsin, Madison |
| Vanderbilt University Medical Center |
| Washington University at St. Louis School of Medicine |
